# Social Facebook With Big Six Approaches for Improved Students’ Learning Performance and Behavior: A Case Study of a Project Innovation and Implementation Course

**DOI:** 10.3389/fpsyg.2020.01166

**Published:** 2020-07-09

**Authors:** Yu-Sheng Su, Hong-Ren Chen

**Affiliations:** ^1^Department of Computer Science and Engineering, National Taiwan Ocean University, Keelung City, Taiwan; ^2^Department of Digital Content and Technology, National Taichung University of Education, Taichung City, Taiwan

**Keywords:** Facebook, Big Six approaches, project innovation and implementation, learning performance, learning behaviors

## Abstract

In Taiwan, classroom lectures are gradually shifting from traditional to diverse digital learning environments through social network websites. Facebook is being used to provide a space for sharing and discussing learning materials and knowledge for teachers and students. In this paper, we focus on the effects of applying Big Six approaches to Facebook on students’ learning performance and behavior in a project innovation and implementation course. The participants were 72 first-year students in a college located in north Taiwan. The experimental participants who took the course were divided into two classes: the experimental group and the control group. While the experimental group used Facebook combined with Big Six approaches, the control group used traditional classroom tools combined with Big Six approaches. The experimental results show that the learning performance and creativity development of students from the experimental group are enhanced after using Facebook with Big Six approaches indicating a great social interaction and discussion cycle. On the other hand, students from the control group were only guided by the teacher. Owing to the lack of interactions between the Internet and the social learning community, there is no obvious enhancement in students’ learning performance and creativity. In addition, we found that the teacher practiced the tips for guiding experimental students to solve the encountered problem, and then the students replied to the classmate’s questions.

## Introduction

The key point of the project innovation and implementation course could boost students’ implementation and creativity development. Teachers do not inculcate knowledge to students any longer, and students are the coordinators of the project (Steffe and Wood, 1990; Lai et al., 2019; Su et al., 2019a). By the time students approach the project actively, they may encounter questions without any great solutions (Wopereis et al., 2008). Through the guidance and explanations of the teacher, students are allowed to understand the problems they face during the project (Macklin, 2008; Gwee, 2008; Chen et al., 2019). Although the guidance of the teacher could lead students to achieve good results in implementing the project, the teacher has to accept that enormous time and effort would be spent on dealing with students’ difficulties, and face-to-face teaching in the classroom might make introvert students shy away from speaking. Additionally, time limitation also widens the distance between the teacher and the students and might eventually result in a lack of sharing and interacting opportunities for the students (Wopereis et al., 2008). Wheatley (1991) mentions that understanding comes from the interactive process with the learning environment; it is through comparing by others and sharing the understanding for implementing knowledge that the cognitive conflict would be processed and the implementation learning would be stimulated.

With the appearance of social networks, the interaction between people, as everyone starts to share their personal information on social network websites, is enhanced. For instance, students learn the lessons on their own by watching lecturing materials that the teacher made beforehand or going over learning materials; subsequently, the students can reflect the problems they encountered during the project. Before the class begins, the teacher gets to know the students’ learning situation, and then, gives suitable objectives based on the students’ schedule. If the teaching effectiveness is enhanced as expected, students’ learning outcomes will be noticeable in the classroom and their learning can be extended after class completion. Social network websites encourage students to learn as a group; their learning motivations are promoted by community learning, sharing knowledge, and the beneficial interaction between community members (Hou et al., 2015).

Chugh and Ruhi (2018) pointed out that Facebook could increase teacher–student and student–student interactions in higher education. Facebook is a representative educational tool. Students can ask questions or share after-class reflections on Facebook anytime and anywhere, and the teacher is also able to respond to students’ questions. Facebook provides more opportunities for students who are extremely shy and introverted to participate in this activity and express their opinions actively (Hou et al., 2009, 2010). Facebook commonly provides a great communicating space for the teacher and students in the class. Assuming that the program of integrating Facebook into the learning community class is well designed, it can be a good method for the students to establish mutual discussions and exchanging learning experiences among their fellows (Garrison et al., 2001). Previous studies indicate that Big Six approaches are useful for combining Facebook into the project courses (Eisenberg and Berkowitz, 1999; Ferguson and Stokes, 2002; Nichols et al., 2005). By using this kind of pedagogy, students’ information technology skills and problem-solving abilities can be cultivated, and the teacher can also learn varied teaching and learning situations that exist in project courses, for example, the interactions and relationships between students and time management, and taking care of students (Evertson and Weinstein, 2006).

In this paper, we explore the effects of applying Big Six approaches to Facebook on students’ learning performance and behaviors in the project innovation and implementation course. A total of 72 college first-years from two classes participated in this experiment; all of them conducted the same learning materials and instructional design. However, the difference between these participants lies in the condition that they are divided into two different learning groups. One is the experimental group that adopts Facebook combined with Big Six approaches, while the other is the control group, which uses traditional classroom tools combined with Big Six approaches. The experiment is conducted in a project innovation and implementation course in a Taiwanese college. We define two research questions while observing this mode. The first research question concerns the differences in learning performance between the experimental group and the control group in the project innovation and implementation course. The second research question evaluates the learning behaviors of the students in the experimental group using sequential analysis.

## Literature Review

### Social Facebook for Education

A social network is a knowledge-sharing platform and a way for a group of people to establish common goals of a project, create collective inquiry action, and expand collective knowledge and skills. The learners are not individuals; they mutually stimulate each other’s learning motivations through the intellectual interaction of community peers (Hou et al., 2015; Chugh and Ruhi, 2018). Facebook can be used as a representative educational tool (Chugh and Ruhi, 2018; Su et al., 2019b), and it has been globally accessible since September 2006. The percentage of Facebook users in Taiwan is extremely high. There are 7.1 million people who log on to Facebook to share their lives or check out news feed. People only need an Internet connection to browse Facebook on their computers or mobile devices. Most people use Facebook at home, and schools are the second most common place where Facebook is used. For those who are actively participating in classes, there are more opportunities for them to demonstrate their thoughts enthusiastically on Facebook (Hou et al., 2009, 2010; Sherry et al., 2011).

Previous studies have indicated the benefits of social network websites for education to increase teacher-student and student-student interactions as well as discussions (Greenhow and Robelia, 2009; Hou et al., 2010, 2015; Hung and Yuen, 2010; Colás Bravo et al., 2013; Chugh and Ruhi, 2018). Researchers have also confirmed the effectiveness of Facebook engagement on students’ learning performance (Hung and Yuen, 2010; Chugh and Ruhi, 2018; Niu, 2019) and social acceptance (Colás Bravo et al., 2013). Greenhow and Robelia (2009) conducted one of the earliest studies to explore the effect of using Facebook on learning. Facebook is used as an accessible educational tool used to facilitate learning experiences. It enhances the interacting opportunities between the teacher and students (Hung and Yuen, 2010), as it attracts most of teachers and students like a strong magnet. Most students use Facebook every day, and students participate and concentrate more in the case of Facebook when compared to the traditional forum (Hou et al., 2015; Chugh and Ruhi, 2018). Sherry et al. (2011) consider Facebook an intensive social interaction since it provides different knowledge, background, and perspective as well as a relaxed social interacting atmosphere. Bowers-Campbell (2008) mentions that Facebook is friendly to students, and it is also student-centered and self-controlled by the student. In addition, the social nature of Facebook is invitation-oriented instead of mandatory participation. Yu et al. (2010) found that using Facebook appropriately helps to share resources and knowledge when students encounter problems during projects and thus, promotes learning effectiveness. Niu (2019) further presented four research directions regarding the adoption of Facebook for academic purposes: an in-depth examination of learning using Facebook, quasi- or true experimental design, the address of potential response bias, and adoption of content analysis.

To sum up, Facebook has commonly been applied to the project innovation and implementation course to provide a great communicative space for the teacher and students. Therefore, the reason we choose Facebook to integrate with the original course is that it offers a convenient and corresponding online learning and interacting pipeline for conducting project courses (Garrison et al., 2001).

### Big Six Approaches for Project

Eisenberg and Berkowitz (1990) came up with the idea of Big Six approaches. The method is applied to the library to promote education and solve information problems. The Big Six approaches are widely known by people, and they have been successfully applied to high schools and universities, namely higher education institutions (Nichols et al., 2005), and collaborative learning for adults (Ferguson and Stokes, 2002). Therefore, Big Six approaches can cultivate students’ information skillsets and the ability to solve problems, and it can allow the teacher to learn of every phenomenon occurring in class (Eisenberg and Johnson, 1996; Evertson and Weinstein, 2006). Applied Big Six approaches are divided into the following six steps.

Step 1: Defining Question: define the question and determine the required information. For example, the teacher guides students to define project objectives and identify issues in order to have a further understanding for the project assignments. This process can be considered to be the clarification of directions of project implementation.

Step 2: Seeking Strategies: ensure the range of information and list the priorities. For example, students are asked to understand the objectives of project implementation *via* collecting information. Students should list out as many project implementation strategies as possible, such as surfing the Internet.

Step 3: Obtaining Information: find and obtain information from the sources. For example, while the students use Web searching engines to look for information, the teacher stays around and helps students find the information by delivering appropriate keywords and ways of searching.

Step 4: Using Information: read and extract the information. For example, during the process of using information, the teacher guides students to find the key parts of information and abstract them.

Step 5: Integrating Information: process and integrate the information. For example, students integrate the assignments into a final project presentation to display their project results.

Step 6: Evaluating the Project: evaluate the completed project and the process. For example, after seeing students’ project results, the teacher gives feedbacks to students so as to improve their project results.

In summary, previous studies find that Big Six approaches are useful for integrating Facebook into project courses (Eisenberg and Berkowitz, 1999; Ferguson and Stokes, 2002; Nichols et al., 2005). In this paper, we combine Big Six approaches with Facebook to enhance students’ project learning effectiveness.

## Methodology

### Participants

In this experiment, 72 first-year students from a college in Taiwan participated. Their average age was 19. All students were divided into two groups: the experimental group comprised 36 students, and the control group consisted of 36 students. Both were taught by the same teacher. All students had to complete the projects given by the teacher and answer the teacher’s questions *via* the collection and explanation of relevant data. The methods of collecting and explaining data were taught by the teacher.

### Learning Materials

The title of the course is “Project Innovation and Implementation.” The learning materials were selected from compulsory courses for first-year students. The goal of the course was to help students learn related techniques and skills for project implementation. The topic of the experiment is “how to apply information technology to solve network congestion problems in online ticketing platforms.” It was designed and exercised by a teacher who had a master’s degree in project courses and had at least 5 years of teaching experience. The topic was based on Big Six approaches to allow students to easily understand the assignments of the project and to find suitable directions.

### Procedure

In this experiment, participants were divided into two groups—an experimental group and a control group—to conduct the experimental procedure. The experiment lasted for 6 weeks, allotting 150 min for each weekly activity. The two groups took their classes at different times. The teacher announced the grading criterion and implemented the regulations of the assignments.

Students from the experimental group employed Facebook combined with Big Six approaches for the experiment while students from the control group applied the traditional classroom tools combined with Big Six approaches. The control group used the six above-mentioned steps.

Finally, students in the experimental group and the control group handed in the project assignments after the experimental activity. The teacher graded the assignments and results according to the project implementation effectiveness.

### Instruments

In Table 1, we revised the assignment evaluation table by Hou et al. (2010) to assess the learning performance of the project. With several teachers from several related fields, including the field of project innovation and implementation education, we can analyze the innovation and implementation assignments of the project and illustrate how to design the learning assessment level of the project. The project assignments are graded by two experts. This study shows that once the Kappa’s reliability value reaches 0.78, formal evaluation can be conducted because the phenomenon means a high degree of consistency and good reliability in the results (Hou et al., 2010). If the explanation of scoring criteria between the raters leads to inconsistency, then amendment or clarification of the scoring definition and the learning assessment level of the project can be done. The grading standard is based on the project scorecard and the same grading standard of every dimension ranges from 0 to 4.0 where 1 means “disagree,” 2 is “average,” 3 is “agree” while 4 is “extremely agree.”

**TABLE 1:** The learning assessment level of the project.

Assessment level	Items
S1. Defining Question	S1-1. Understanding the project requirement clearly
	S1-2. Knowing the core goal of the project
	S1-3. Being able to bring out related implementation issues
S2. Seeking Strategies	S2-1. Being able to come up with strategies for looking for implementation information
	S2-2. Define information sources for searching
	S2-3. Ascertain the importance of information
	S2-4. Knowing numerous implementing ways of grasping information
	S2-5. Using appropriate standards to select information sources (such as authoritative, liquidity, availability, legibility, range and format.)
S3. Obtaining Information	S3-1. Being able to obtain implementation information independently
	S3-2. Being able to obtain implementation information by query
	S3-3. Being able to obtain information by getting access to the Internet
	S3-4. Being able to obtain information by a suitable pipeline
S4. Using Information	S4-1. Being able to abstract main data correctly
	S4-2. Being able to read, hear, or observe implementation information carefully
	S4-3. Being able to cite the resources correctly
	S4-4. Being able to identify the truth and the opinions
S5. Integrating Information	S5-1. Being able to sort out data correctly
	S5-2. Being able to organize the messages properly
	S5-3. Being able to present the project implementation results
	S5-4. The display of graphics and text (for example, whether the content is logical, and the graphics are coherent.)
	S5-5. Being able to integrate multiple information
S6. Evaluating the Project	S6-1. Being able to self-evaluation the advantages and disadvantages of the project
	S6-2. Being able to bring up effective implementation ways of improving the work
	S6-3. Being able to self-assess the degree of completion
	S6-4. Being able to evaluate every phase during completing the project.

### Data Collection and Analysis

By carefully observing the students, the teacher mostly learned about the students’ project execution and learning situations. Students use various social tools for communication and cooperation, which inhibit the teacher from monitoring the students’ learning processes, giving them timely guidance, and evaluating their personal contributions correctly. Based on the Graph API of Facebook, we aggregated Facebook logs, which include content, writer, time, replying posts, number of likes, number of shares, community titles, number of members, links, and image hyperlinks. Subsequently, the log data was stored in our database to determine every student’s project execution and learning situation (Hung et al., 2020).

Bakeman and Gottman (1997) introduced the sequential analysis method to point out how to select every detail and every specific type for different research scenarios during the discussing and observing process. It somehow illustrates what kind of statistical method should be used in the experiment. According to Bakeman and Gottman (1997), the sequential analysis method is more useful in inferring the overall sequence of students’ project executing and learning process. The sequential relationships in the behavioral modes can reach statistical significance. Gunawardena et al. (1997) propose the Interaction Analysis Model (IAM). Interaction Analysis Model is useful for analyzing the project executing and learning process of students, and it increases the effectiveness of quantitative content analysis. Since 1997, IAM has been an important sequential analysis methods for students’ learning behaviors. Sequential analysis aims to find meaningful learning patterns in the process of doing the project for students (Bakeman, 1986). Those learning patterns can be challenging or controversial, which provides different perspectives. We apply GSEQ (Generalized Sequential Querier), a common computer statistics software usually used by researchers to perform an analysis such as ours (Bakeman and Gottman, 1997). GSEQ supports the work of analyzing the logs data of Facebook, also, it lowers the complexity of behavioral coding.

Based on Hou et al. (2010), we induct the similarities in the study to form coding behaviors for project learning. The effective data points on students’ project execution and learning process, and the teacher’s suggestions about the experiment were excluded from the data. Cohen’s kappa coefficient is a statistical method for the correspondence between the classification codes. When the kappa coefficient is not less than 0.7, it shows a high degree of consistency and good faith can be achieved. Two experts, who had more than 3 years of teaching experience in the course, conducted the training of behavioral coding. Subsequently, the common score was set under the coding table for the learning behavior of students. According to the students’ project execution and learning process, the coding unit concludes the original message and its subsequent replies by the other fellows. The sequence of the coding table is shown in Table 2.

**TABLE 2:** The coding behaviors for the project.

Coding	Stage	Explanations
S1	Defining Question	Understanding the project implementation goals and issues from the assignments clearly, as well as the requirements of implementation clearly.
S2	Seeking Strategies	Being able to describe the sources of required implementation information.
S3	Obtaining Information	Obtaining relevant information and then conducting inquiry of project implementation.
S4	Using Information	Abstracting all the proper project implementation information.
S5	Integrating Information	Integrating and illustrating all proper information for implementation and prospective into the work.
S6	Evaluating the Project	Evaluating project results and give reflections for improving project implementing procedures.

## Results

### Analysis of Project Learning Performance

In this experiment, the experimental and control groups practiced under the learning assessment level of the project. We revised this level by Hou et al. (2010) to be the basis of grading project learning achievements. The learning assessment level is divided in the six following categories: defining questions, seeking strategies, obtaining information, using information, integrating information, and evaluating the project.

The experimental results show that there are 72 final projects in total, which come from the experimental group and the control group. Two experts were hired to grade the students’ projects. Before assessing, we conducted training for the two experts to allow the scorers to understand scoring criteria and to grade students in both groups. If there is any inconsistency between the raters’ grading, the factors causing the inconsistency need clarification. If there is any inconsistency between the raters’ explanations of grading, the raters must inspect scoring criteria more closely and adjust their grading. According to Kappa’s reliability analysis, we found that the Kappa rate of the control group (Kappa rate = 0.829, *p* = 0.000 < 0.05) and the experimental group (Kappa rate = 0.811, *p* = 0.000 < 0.05) both reached more than 0.8 in reliability; thus, the two experts showed high degrees of consistency and good reliability.

After the experts evaluated the students’ project, it was found that the average score of the experimental group (mean = 3.529, SD = 0.650) was higher than that of the control group (mean = 2.863, SD = 0.824). Thus, we use the paired-samples *t*-test to show the differences between the learning performances of the two groups. The results of the paired samples *t*-test for the two groups’ learning performance are shown in Table 3. The value of the *t*-test was -5.132 (*p* < 0.01). This result indicates a significant difference between the learning performances of the two groups. Further comparison can be conducted to determine whether there are any differences in the sub-items of the project learning assessment level of the two groups in Table 4.

**TABLE 3:** Paired-samples *t*-test for two groups’ learning performance.

Group	*N*	Mean	SD	*t*	*p*
Experiment group	36	3.529	0.651	−5.132	0.000**
Control group	36	2.863	0.824		

**p < 0.01.

**TABLE 4:** Paired-samples *t*-test for two groups’ project learning assessment levels.

Assessment level	Items	Group	Mean	SD	*t*	*p*
S1. Defining Question	S1-1	Experiment group	3.740	0.541	6.937	0.000**
		Control group	2.222	0.902		
	S1-2	Experiment group	4.000	0.126	1.000	0.328
		Control group	3.961	0.209		
	S1-3	Experiment group	3.963	0.209	12.949	0.000**
		Control group	2.872	0.344		
S2. Seeking Strategies	S2-1	Experiment group	2.301	0.635	1.775	0.083
		Control group	2.003	0.522		
	S2-2	Experiment group	3.912	0.417	1.813	0.080
		Control group	3.524	0.947		
	S2-3	Experiment group	3.481	0.665	4.985	0.000**
		Control group	2.262	0.964		
	S2-4	Experiment group	2.134	0.694	1.082	0.285
		Control group	1.911	0.668		
	S2-5	Experiment group	2.352	0.775	4.268	0.000**
		Control group	1.521	0.511		
S3. Obtaining Information	S3-1	Experiment group	3.964	0.209	1.596	0.122
		Control group	3.741	0.619		
	S3-2	Experiment group	2.224	1.126	–1.622	0.112
		Control group	2.742	1.054		
	S3-3	Experiment group	3.874	0.626	0.640	0.526
		Control group	3.742	0.752		
	S3-4	Experiment group	3.634	0.212	1.517	0.103
		Control group	3.482	0.994		
S4. Using Information	S4-1	Experiment group	3.961	0.209	2.098	0.045*
		Control group	3.701	0.559		
	S4-2	Experiment group	3.654	0.573	6.834	0.000**
		Control group	2.351	0.714		
	S4-3	Experiment group	3.814	0.288	2.471	0.038*
		Control group	3.413	1.146		
	S4-4	Experiment group	3.834	0.388	10.732	0.000**
		Control group	2.300	0.559		
S5. Integrating Information	S5-1	Experiment group	3.913	0.288	2.980	0.406
		Control group	3.772	1.154		
	S5-2	Experiment group	3.572	0.590	0.668	0.508
		Control group	3.431	0.728		
	S5-3	Experiment group	4.000	0.126	1.000	0.328
		Control group	3.961	0.209		
	S5-4	Experiment group	3.434	0.728	0.236	0.814
		Control group	3.390	0.499		
	S5-5	Experiment group	3.784	0.422	2.483	0.319
		Control group	3.561	0.915		
S6. Evaluating the Project	S6-1	Experiment group	3.742	0.541	9.138	0.000**
		Control group	1.700	0.926		
	S6-2	Experiment group	2.784	1.166	4.701	0.000**
		Control group	1.353	0.885		
	S6-3	Experiment group	3.093	1.164	6.641	0.000**
		Control group	1.261	0.619		
	S6-4	Experiment group	3.964	0.209	5.662	0.000**
		Control group	2.741	1.010		

*p < 0.05, **p < 0.01.

In the sub-items of the learning assessment level, within the stage of Defining Question (S1), the two groups show a significant difference between S1-1 and S1-3. Students in the experimental group had higher average scores on S1-1 and S1-3 than those in the control group, which showed that students in the experimental group were more eager to ask questions and discuss, and Facebook helped to understand the implementation goals of the project. Most students in the control group conducted the experiments individually, and one of the students said, “Through the discussion, I realize that I may be confined to an individual problem due to the information that, I find, tend to be one-way.”

In the stage of Seeking Strategies (S2), the two groups showed a significant difference in S2-3 and S2-5. The experimental group had a higher average score on S2-5 than the control group, which showed that utilizing Facebook for discussion and instruction helped students in the experimental group determine the importance of related information and to adopt suitable references.

In the stage of Using Information (S4), the two groups showed a significant difference in S4-2 and S4-4. The experimental group had a higher average score on S4-4 than the control group, which indicated that using the social functions of Facebook was helpful for students in the experimental group to distinguish between facts and opinions. Students in the experimental group were able to read, hear, and observe information much more carefully than those in the control group.

In Evaluating the Project (S6), the two groups showed notable differences in S6-1, S6-2, S6-3, and S6-4. Students in the experimental group had a higher average than those in the control group, which indicated that combining Big Six approaches with Facebook significantly enhanced the students’ project learning achievements.

### Analysis of Project Learning Behaviors for the Experimental Group

During the experiment, we collected data from the experimental group. The total number of Facebook log data is 632; however, 26.8% of them include unrelated topics. There are numerous factors on Facebook that distract students from the discussions, for example, sociability, entertainment, games, messages from the teacher for instruction and responses, and incomplete posts and replies. After filtering out useless data, there were 436 effective feedback sheets in total. According to the coding table for the project learning behavior, Facebook log data are divided into six categories. There are 75 messages in Defining Question (S1), which accounts for 17.2% of the total. The number of messages in the other stages are as follows: Seeking Strategies (S2) = 46 (10.5% of the total); Obtaining Information (S3) = 54 (12.4% of the total); Using Information (S4) = 105 (24% of the total); Integrating Information (S5) = 67 (15.4% of the total); and Evaluating the Project (S6) = 89 (20.4% of the total).

**FIGURE 1:**
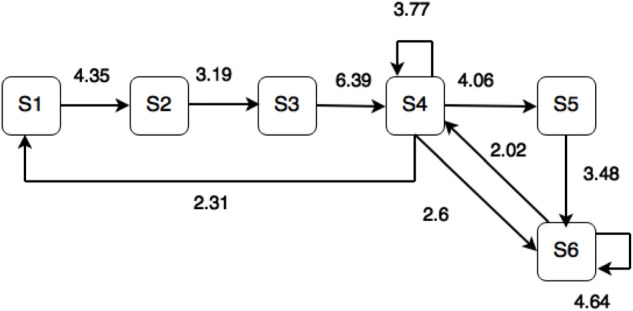
The learning behaviors of the experimental group.

We used the sequential analysis method to classify students’ learning patterns. According to classification by sequential analysis, there are 463 codes in students’ learning behaviors in total. The codes for Defining Question (S1) account for 17.2% of the total. The codes for Seeking Strategies (S2) account for 11% of the total. The codes for Obtaining Information (S3) account for 12% of the total. The codes for Using Information (S4) account for 23.5% of the total. The codes for Integrating Information (S5) account for 15.1% of the total. The codes for Evaluating the Project (S6) account for 21.2% of the total. According to the inter-rater reliability proposed by Landis and Koch (1977), Facebook log data are given to the other rater to ensure consistency during the coding process, and the Kappa coefficient is 0.85^∗∗∗^(*p* < 0.001), which has achieved significance. This study transfers the standard codes into the transition diagrams, and the direction of the arrow points from the start encoding to the target encoding. The numbers on the line represent the conversion behavior of the z-score. As Table 5 shows, the sequential analysis method is used to calculate every sequential z-score. If the z-score is greater than 1.96, it indicates that the statistics have achieved significance (Bakeman and Gottman, 1997). Finally, as Figure 1 shows, the sequential relational analysis graph, in which the significance lies in the coding levels, is drawn based on the results.

**TABLE 5:** The sequential analysis result of the experimental group.

	S1	S2	S3	S4	S5	S6
S1	0.27	4.35*	–0.32	–0.90	–2.07	–0.61
S2	–0.61	–3.23	3.19*	–2.07	–2.65	–2.36
S3	–1.48	–2.65	–2.36	6.39*	–2.36	–2.94
S4	2.31*	–2.94	–0.61	3.77*	4.06*	2.60*
S5	–1.77	–2.07	–2.94	0.27	1.43	3.48*
S6	1.73	–1.19	–2.36	2.02*	–0.02	4.64*

*p < 0.05.

In the experiment, we conducted the sequential analysis method to examine the behavioral transfer from S1 to S6 for understanding experimental students’ learning behaviors using Facebook combined with Big Six approaches. As shown in Figure 1, when the core issue of the project is divided into relevant small issues (S1), the processes of seeking multiple information resources and judging important assignment resources (S2) are started. Subsequently, useful information is obtained through assignment resources (S3) followed by reading the details, discussing them, and distinguishing the accuracy (S4). Students integrate proper ideas and solutions for completing the assignment work (S5). Finally, students self-evaluate their accomplishments (S6).

In analyzing behaviors, we found that students interact with each other more in S4. Moreover, the behavioral relationship between S4 and S6 is mutual; thus, it is known that students integrate the proper ideas and solutions information during the stage of Using Information (S4). From the behavioral transfer S4- > S5- > S6, we find that students look up Facebook to get possible implementing methods and evaluate the feasibility of the methods. If students encounter some problems, they will go back to look up related information to revise the implementation and project presentation. It can be seen from the behavioral transition that there are possibilities for being trapped in the stage of Using Information (S4) and the stage of Defining Question (S1). From the interview, it can be seen that students are likely to have questions after conceiving doubts and that some topics that are unrelated to the projects may be raised. However, by going back to the topic of the experimental activities, applying proper implementing ideas and methods to the projects, and the guidance of the teacher and classmates, students can return to Using Information (S4). In that stage, we found that students carefully read, hear, or observe project-related information. With assistance from the teacher and partners on Facebook, they can get the correct information and provide their partners with a reliable information source. Additionally, the reply and remind function of Facebook is commonly used among students; although ideas that are posted may not be adopted instantly, students still share possible information and their opinions about those ideas.

In addition, we found that students do fewer searches for information (S3); instead, they spent more time reading carefully to distinguish between facts and the author’s opinions. From the interview, a student said that “The variety of information is given priority during information collection. Subsequently, we conduct an inter-examination of the collected information. In fact, questions and information proposed by other classmates during the discussion on Facebook have really high reference value, which allows me to get the whole picture in a short time. I save a lot of time searching on Google, which makes the inter-examination of the accuracy of information much easier.” Therefore, students spent little time searching for information (S3); instead, they spent much more time carefully choosing to identify facts and opinions.

As for Defining Question (S1) and Seeking Strategies (S2), a student shared that “The result of the Facebook discussion was based on everyone’s opinion. First, the range of defining questions had not been clarified, and we primarily adopted some members’ opinions, deciding to search on Google with certain keywords.” What the student said showed that students tended to transfer from Defining Question (S1) to Seeking Strategies (S2) once they received advice about searching keywords related to solving the project problems, which usually led to disappointing results or wrong directions. Students try various keywords on Google to look for a suitable way to work out their project problems. They share possible answers on Facebook to allow others to obtain useful information to accomplish the project as soon as possible. The behaviors mentioned above continued to repeat during the experiment, which might have been caused by the social services of Facebook. We suggest that the teacher and partners on Facebook give students positive feedback after delivering memory feedback.

## Conclusion and Discussion

In this paper, we explore how combining Big Six approaches with Facebook improves students’ project learning performance. We analyzed the differences in project learning performance between the experimental and control groups. The teacher adopted the Big Six approaches to guide students in both groups to find possible solutions and directions using different instructional tools for the course, “project innovation and implementation.” We found that there are significant differences between both groups. The results indicate that students in the experimental group had a higher average score than those in the control group. Further discussion of combining the Big Six approaches with different instructional tools in both groups should be done to show different project learning outcomes. We found that there are significant differences in Defining Question (S1), Seeking Strategies (S2), Using Information (S4), and Evaluating the Project (S6).

On S1-1 and S1-3, the experimental group obtained higher average scores than the control group. It indicates that it is useful for the experimental group to understand the goals and requirements of the project using Facebook to conduct the experiment. We found that on S2-3 and S2-5, the experimental group scored higher than the control group.

In the experimental group, the findings show that the students’ cooperation with the teacher and classmates can help in judging the importance of information and knowing how to use them appropriately. On S4-2 and S4-4, the experimental group had higher average scores than the control group. It indicates that using Facebook helps students carefully observe information and distinguish facts from opinions. The experimental group received higher average scores than the control group on every sub-item in S6. It shows that students share related information about the project and give their feedback and suggestions on Facebook to help their classmates improve their projects. Moreover, the teacher equipped the students with the ability to solve problems. Students can come up with ideas about project innovation and learning and share their ideas with others on Facebook to accomplish the projects.

In addition, we used the sequential analysis method to demonstrate students’ project learning behaviors of combining Big Six approaches with Facebook. The results show that there are more interactions in S4 than in the other stages. There is a two-way relationship between S4 and S6. This finding shows that students use Facebook to share a large number of search results, and they can find useful information to solve project-related problems with other peers’ feedback. It is shown in the transition diagram of Defining Question (S1) that students will further raise issues based on their doubts. In addition, some topics unrelated to the projects may be raised. However, by going back to the topic of the experimental activities, applying proper implementing ideas and methods to the projects, and the guidance of the teacher and classmates, students can return to the stage of using information (S4). Students not only raise questions during discussions on Facebook but also do self-learning when answering their questions. By answering the questions, students further put forward their memory knowledge; memory knowledge leads students to explain and illustrate knowledge with their thoughts.

In summary, from students’ project learning performance and learning behaviors, we found that students in the experimental group, who applied Facebook to their learning, created a great peer interaction and discussion and conducted more learning-related behaviors. Therefore, we demonstrate that combining Big Six approaches with Facebook helps improve the quality of the course, “project innovation and implementation.”

## Data Availability Statement

All datasets presented in this study are included in the article/supplementary material.

## Ethics Statement

Ethical review and approval was not required for the study on human participants in accordance with the local legislation and institutional requirements. Written informed consent from the college students/participants was not required to participate in this study in accordance with the national legislation and the institutional requirements.

## Author Contributions

Both authors contributed equally to the conception of the idea, implementing and analyzing the experimental results, and writing the manuscript and read and approved the final manuscript.

## Conflict of Interest

The authors declare that the research was conducted in the absence of any commercial or financial relationships that could be construed as a potential conflict of interest.
